# Unraveling mitochondria‐targeting reactive oxygen species modulation and their implementations in cancer therapy by nanomaterials

**DOI:** 10.1002/EXP.20220115

**Published:** 2023-04-05

**Authors:** Haibao Peng, Feibai Yao, Jiaxu Zhao, Wei Zhang, Lingchao Chen, Xin Wang, Peng Yang, Jing Tang, Yudan Chi

**Affiliations:** ^1^ Department of Neurosurgery, Huashan Hospital, Institute for Translational Brain Research, State Key Laboratory of Medical Neurobiology, MOE Frontiers Center for Brain Science Fudan University Shanghai China; ^2^ Engineering Research Center of Molecular‐ and Neuro‐imaging of Ministry of Education, School of Life Science and Technology Xidian University Xi'an Shaanxi China; ^3^ Department of Materials Science and Engineering Stanford University Stanford California USA

**Keywords:** cancer therapy, mitochondria‐targeting, nanomaterials, reactive oxygen species

## Abstract

Functional subcellular organelle mitochondria are emerging as a crucial player and driver of cancer. For maintaining the sites of cellular respiration, mitochondria experience production, and accumulation of reactive oxygen species (ROS) underlying oxidative damage in electron transport chain carriers. Precision medicine targeting mitochondria can change nutrient availability and redox homeostasis in cancer cells, which might represent a promising strategy for suppressing tumor growth. Herein, this review highlights how the modification capable of manipulating nanomaterials for ROS generation strategies can influence or compensate the state of mitochondrial redox homeostasis. We propose foresight to guide research and innovation with an overview of seminal work and discuss future challenges and our perspective on the commercialization of novel mitochondria‐targeting agents.

## Graphical abstract

Functional subcellular organelle mitochondria are emerging as a crucial player and driver of cancer. Precision medicine targeting mitochondria can change nutrient availability and redox homeostasis in cancer cells. This review highlights how the modification capable of manipulating nanomaterials for reactive oxygen species (ROS) generation strategies can influence the rate of mitochondrial ROS production and their implementations in cancer therapy.

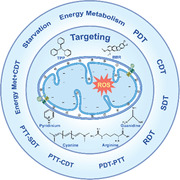

## 1 INTRODUCTION

**FIGURE 1:**
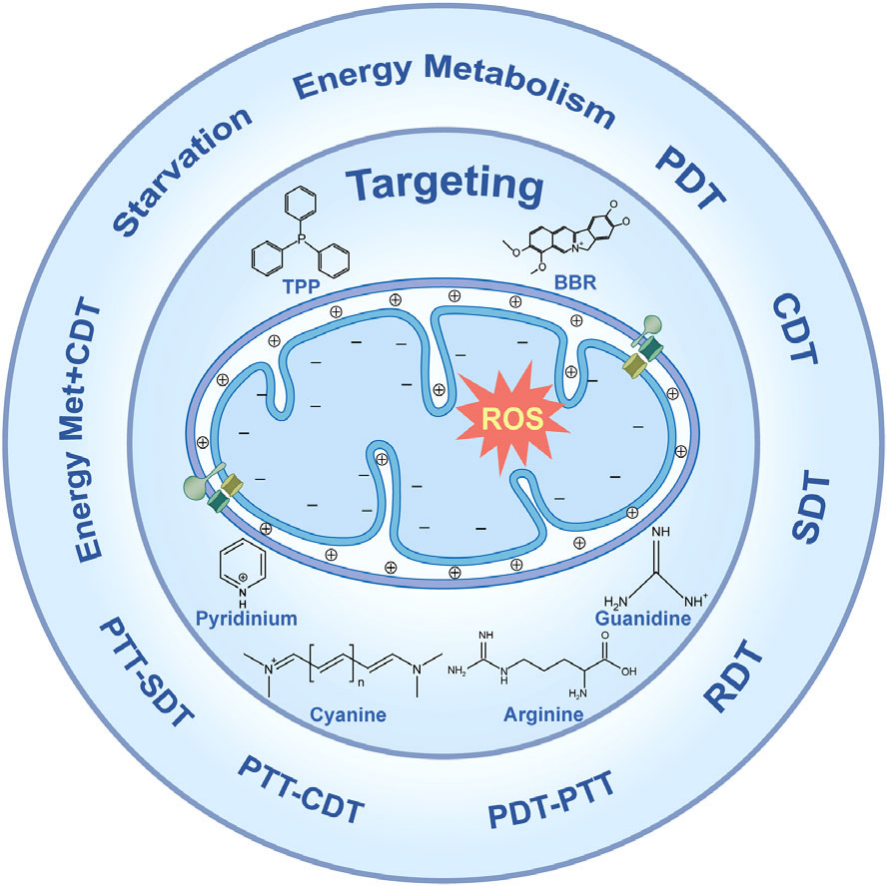
The current toolbox of mitochondria‐targeting‐based reactive oxygen species (ROS) modulation (photodynamic therapy (PDT), chemodynamic therapy (CDT), sonodynamic therapy (SDT), radiodynamic therapy (RDT), photothermal therapy (PTT), starvation strategy, and the combination methods) for cancer treatment. Positively charged molecules (triphenylphosphonium (TPP), berberine (BBR)) and hydrophobic natural amino acid (phenylalanine, tyrosine, isoleucine) are presented as alternative sources to target mitochondria.

The term “mitochondrion” originated from the Greek words “*mitos*” and “*chondrion*”, which means thread and granules‐like.^[^ ^1^ ^]^ The morphology of mitochondria is a rod‐shaped structure in the range from 0.5 to 1 μm.^[^ ^2^ ^]^ As shown in Figure 1, mitochondria have an outer and inner membrane, a kind of gel‐like matrix and intermembrane spaces. The outer membrane is anchored with proteins known as porins, which allow movement of ions and permit peptides into and out of the mitochondrion. The folded inner membrane forms cristae which contain many electronic transport chains for redox reactions from nicotinamide adenine dinucleotide (NADH) to oxygen. Since an electrochemical gradient across the membrane pumping protons out of the matrix contributes to creating negative charge of inner membrane. These structures of mitochondria are quite unusual for an intracellular organelle.

As the powerhouse of the cells, mitochondria not only produce energy, but also take part in cell differentiation, cell signaling, and cell death. With the adenosine triphosphate (ATP) production,^[^ ^3^ ^]^ mitochondria also refer to the transition of metabolites,^[^ ^4^, ^5^, ^6^ ^]^ and generation of redox molecules,^[^ ^7^ ^]^ which inevitably produce reactive oxygen species (ROS).^[^ ^8^ ^]^ Mitochondrial‐derived ROS (H_2_O_2_, O_2_^•−^, •OH, ^1^O_2_) are the products of the oxygen metabolism, which play vital roles as regulatory molecules at physiologic levels. Various physiological reactions, including electron transport by the electron transport chain and NAD(P)H oxidases are the main producers of ROS in mitochondria.^[^ ^8^ ^]^ In normal cells, the multiple antioxidant pathways neutralize toxic ROS and maintain their redox homeostasis, such as glutathione (GSH) and superoxide dismutase. And abnormal level ROS can damage mitochondria or even induce cell death. On the contrary, cancer cells survive within a higher level of mitochondrial‐derived ROS, due to their metabolic demand for production of biomass and energy.^[^ ^7^ ^]^ Therefore, it is promising that targeting mitochondria and intervening mitochondrial‐derived ROS level might be an effective method to trigger cancer cell death.

Advances in nanotechnology are deeply intertwined with “nanocatalytic medicine,” ROS‐based cancer therapy has been developed in conjunction with other nanocatalytic‐therapeutic modalities. For example, chemodynamic therapy (CDT) is a kind of tumor microenvironment (TME)‐activated localized Fenton catalytic reaction for cancer therapy.^[^ ^9^, ^10^, ^11^, ^12^, ^13^, ^14^ ^]^ Besides, photodynamic therapy (PDT)^[^ ^17^, ^18^, ^19^, ^20^, ^21^, ^22^ ^]^ and sonodynamic therapy (SDT)^[^ ^13^, ^15^, ^16^ ^]^ lie beneath intense investigations for ROS‐based cancer therapy. However, inefficient ROS generation and low efficiency of ROS limit its further bio‐applications.^[^ ^17^ ^]^ Recently, subcellular organelle‐targeting, especially that mitochondria‐targeting has attracted great attention in ROS‐based cancer therapy.^[^ ^18^ ^]^

**FIGURE 2:**
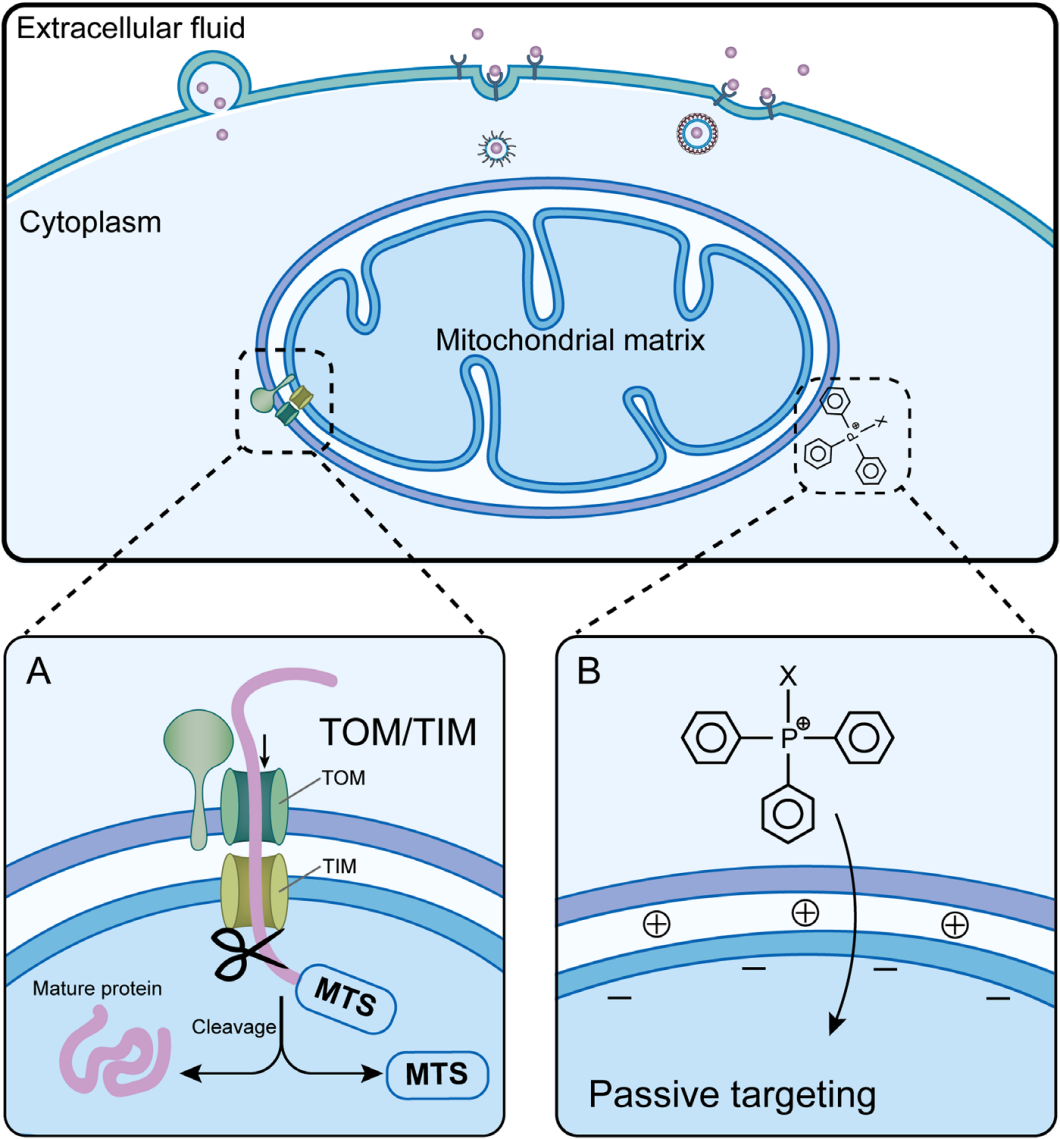
Biological pathways for mitochondria‐targeting.^[^ ^19^ ^]^  (A) Mitochondria‐targeting sequences (MTS)‐mediated protein‐import strategy. Translocation of mitochondrial proteins from cytoplasm to mitochondrial matrix requires the guidance of MTSs and assistance of transmembrane proteins, such as translocase of the outer membrane (TOM) complex and translocase of the inner membrane (TIM) complex. Once MTSs are recognized and transported to cytoplasm through inner membrane, peptidases cleave MTSs to irreversibly anchor the peptide chain, followed by ATP‐dependent inward transportation. (B) Mitochondrial membrane potential (MMP)‐mediated passive targeting strategy. Mitochondria tropic molecules, such as lipophilic cations (triphenylphosphonium, TPP^+^) pass directly through inner membrane of mitochondria driven by the high transmembrane potential, serving as a conjugative carrier of bioactive molecules.

In this review, we discuss how to design the appropriate nanomaterials and nanostructures for ROS production (Figure 1). We then introduce the mitochondria‐targeting features and strategies that have been used to improve their accumulation efficacy through mitochondrial membrane potential (MMP)‐mediated passive‐targeting pathway or through mitochondria‐targeting sequences (MTS)‐mediated protein‐import pathway (Figure 2 ). We also highlight the advances wherein unique functions of ROS regulation strategies can be used for improving traditional mitochondria‐targeting cancer therapy. Finally, we share our perspective on the challenges and outlook of the application of nanomaterials toward cancer treatment through mitochondria‐targeting ROS modulations.

## 2 MITOCHONDRIA‐TARGETING STRATEGY

Mitochondrial outer membrane porins structure and negative charge properties of inner membrane are the rationale for mitochondria‐targeting strategies.^[^ ^19^ ^]^ And there are two patterns for mitochondria‐targeting (Figure 2): (1) the MTS‐mediated protein‐import strategy, and (2) MMP‐mediated passive targeting strategy. The above two strategies are the basic principles for designing mitochondria‐targeting nanomaterials, which would be promising to enhance the therapeutic efficacy of ROS‐based cancer therapy.

### 2.1 MTS‐mediated protein‐import strategy

MTS strategy benefits from mitochondrial porins structure, where natural biomolecule (amino acid or peptide) is delivered from outer membrane into inner membrane. The TOM/TIM (translocase of the outer membrane/translocase of the inner membrane) complexes are the effector protein of MTS strategy, which translocate proteins for oxidative phosphorylation. Some new strategies have been developed to reach mitochondria through TOM/TIM complexes. For example, Cui et al. proposed a conjugative dual peptide method to effectively deliver dye 5‐FAM (5‐carboxyfluorescein) into mitochondria.^[^ ^20^ ^]^ By conjugating cell penetrating peptide and MTS into one single molecule, the results demonstrate considerable benefits of this strategy, including an unexpected, synergistic effect of the secondary structure of conjugated peptides for mitochondria internalization. Meanwhile, their research raises important points into the rational design of MTS‐mediated mitochondria uptake. Naturally, MTS sequences have amphipathic helix structures. Lee et al. systematically designed and synthesized a series of amphipathic peptides.^[^ ^21^ ^]^ They found that two peptides with cyclohexyl showed high mitochondria localization, and without significant adverse effects, which provide insights into robust targeted delivery of bioactive compounds. However, it is worth noting that the protection of peptides from enzymatic cleavage is an important concern for peptides‐related targeting strategy.

### 2.2 MMP‐mediated passive targeting strategy

MMP‐mediated targeting method is a kind of passive targeting strategy based on negative‐positive electrostatic interactions. In normal cells, the MMP is at the values between −100 and −140 mV,^[^ ^22^ ^]^ however, the MMP of cancer cells present much lower (∼ −180–−220 mV) than that of normal cells due to high metabolism demand for production of several biomacromolecules.^[^ ^22^ ^]^ Thus, design of necessary positively charged molecules or nanomaterials through MMP‐mediated strategy can effectively target mitochondria (Figure 2). Typically, triphenylphosphonium‐based (TPP^+^) modification is the main mitochondria‐targeting method, and the routine way is linking TPP^+^ with bioactive molecules or nanomaterials to deliver the imaging probes, antioxidants, and drugs to mitochondria. Jung et al. conjugated the near‐infrared (NIR)‐absorbing cryptocyanine probe with TPP molecule for mitochondria‐targeting imaging and phototherapy.^[^ ^23^ ^]^ Additionally, Cheng et al. reported a series of TPP^+^‐conjugated bioactive molecules like the analogs of metformin,^[^ ^24^ ^]^ and many fine‐tuned mitochondrial‐targeted cationic compounds are synthesized to strengthen anti‐proliferative and radio‐sensitizing effects for pancreatic cancer cells.

It should be noted that other lipophilic cations, such as pyridinium, rhodamine, and cyanine derivatives have been verified to colocalize within mitochondria.^[^ ^25^, ^26^, ^27^, ^28^ ^]^ Compared with these cations’ molecules, TPP^+^‐conjugated platforms take quite many advantages including the stability of targeting units, the relatively simple technology of synthesis and purification, and the low specificity on another subcellular organelle. Furthermore, the typically positively charged (arginine, lysine) and hydrophobic (phenylalanine, tyrosine, isoleucine) natural amino acid and synthetic peptides are presented as alternative sources to target mitochondria. Consequently, functional modification with charged natural amino acids and peptides is also a possible method for mitochondria‐targeting (Figure 2).

## 3 NANOMATERIALS FOR MITOCHONDRIA‐TARGETING ROS MODULATION

### 3.1 Nanomaterials for ROS modulation

Catalytic Nanomaterials (nanozymes,^[^ ^29^, ^30^, ^31^ ^]^ photocatalysts^[^ ^32^, ^33^ ^]^ and sonocatalysts^[^ ^34^, ^35^ ^]^) have been applied to produce toxic ROS, as for instance iron‐based nanomaterials, which transfer hydrogen peroxide (H_2_O_2_) to hydroxyl radical (•OH) through Fenton catalytic reaction. Due to the elevated level of aerobic glycolysis, cancer cells are much more sensitive to glucose level. Delivering glucose oxidase (GOx) through nanostructures could consume the glucose in cancer cell, and this catalytic reaction will simultaneously provide H_2_O_2_.^[^ ^36^, ^37^ ^]^ Besides, nanostructure‐based photosensitizers,^[^ ^38^, ^39^, ^40^, ^41^, ^42^, ^43^ ^]^ sonodynamic agents,^[^ ^13^, ^15^, ^16^ ^]^ and nanocatalytic agents^[^ ^27^, ^28^, ^29^, ^30^, ^31^ ^]^ are regarded as effective approaches to generate representative ROS (^1^O_2_, •OH, O_2_^•−^). For example, ZIF‐8 derived carbon nanoparticle is a photosensitizer, which could absorb light energy to generate singlet oxygen (^1^O_2_) by energy transfer. In all, nanomaterials play an important role in ROS generation and modulation, and show great potential for bio‐applications.

### 3.2 Mitochondria‐targeting by modification strategy

Nowadays, subcellular‐targeting ROS modulation has achieved great triumphs,^[^ ^44^, ^45^ ^]^ which can increase therapeutic effect and reduce side effects. As the major subcellular organelle, mitochondria are important targets to enhance ROS production efficiency.^[^ ^15^, ^16^, ^36^, ^42^, ^46^, ^47^, ^48^, ^49^ ^]^ Meanwhile, the diversity of surface‐active functional groups (such as −NH_2_, N_3_, or −COOH),^[^ ^50^, ^51^, ^52^, ^53^, ^54^ ^]^ tunable structure of nanomaterials and responsive methods^[^ ^55^, ^56^, ^57^ ^]^ also provide powerful tools for mitochondria‐targeting modification strategy. Yang group constructed magnetic composite nanoparticles (MMCNs) as a mitochondria‐targeting photothermal agent for cancer treatment, which manifests high tumor accumulation and excellent tumor regression by hyperthermia and the cytotoxic ROS.^[^ ^58^ ^]^ Notably, Liang group reported a mitochondrial oxidative stress amplifier for HepG‐2 tumor and hepatic PDX tumors therapy, which benefits from the mitochondria targeting ability of TPP modification.^[^ ^59^ ^]^ Thus, mitochondria‐targeting by surface modification strategy could be an effective way for precision subcellular‐targeting ROS modulation.

## 4 MITOCHONDRIA‐TARGETING‐BASED NANOMATERIALS FOR CANCER TREATMENT

**FIGURE 3:**
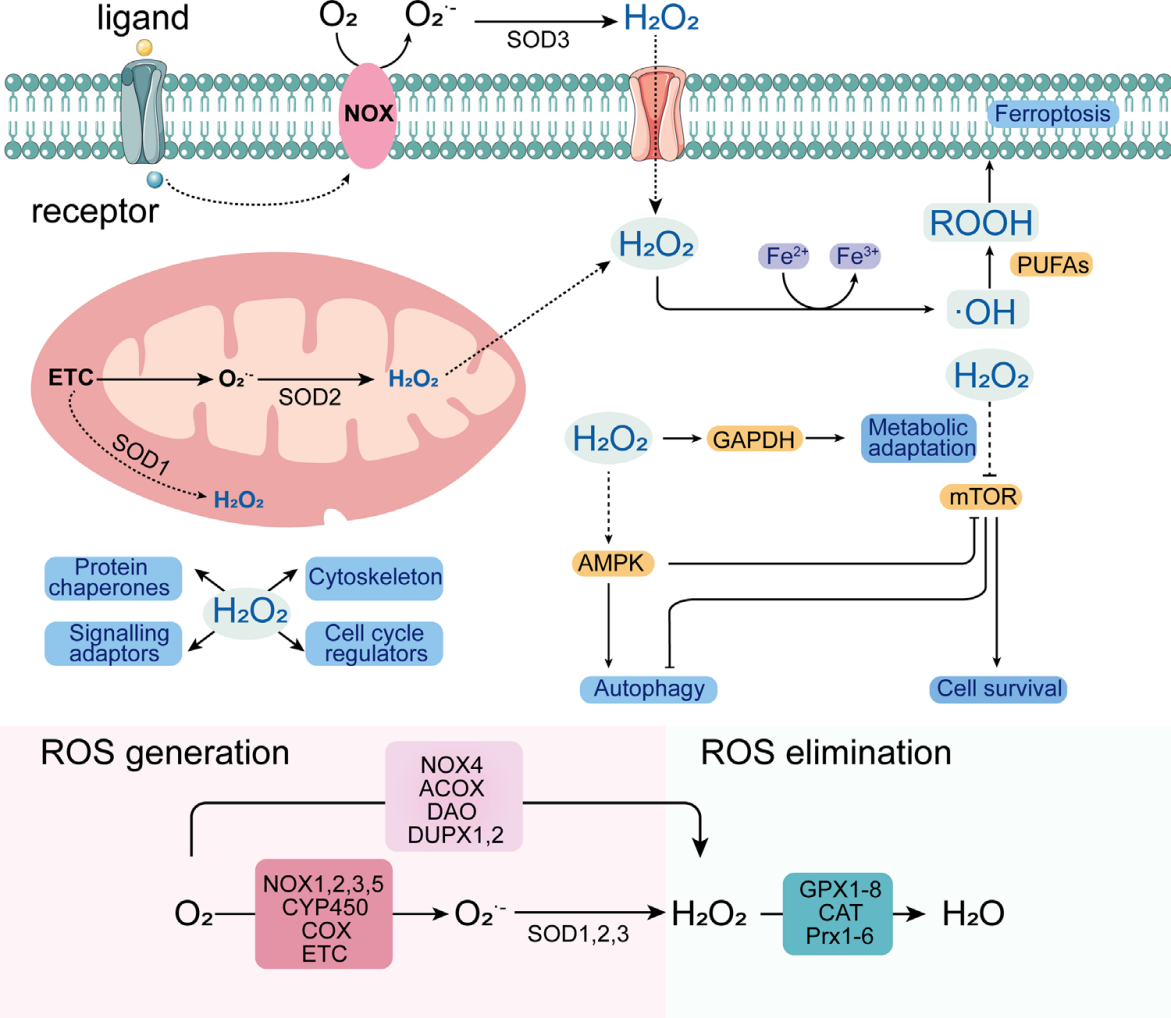
Mitochondria sources reactive oxygen species (ROS) (O_2_^•−^, H_2_O_2_, •OH, etc.) within the cell, which yield diverse biological activities (pale blue) as cellular targets. Examples of enzymatic systems that are responsible for ROS production and ROS elimination. Reproduced with permission.^[^ ^60^ ^]^ Copyright 2021, Elsevier. Reproduced with permission.^[^ ^68^ ^]^ Copyright 2020, Springer Nature.

Mitochondria ROS derive from various sources. As Figure 3 shown, mitochondria sources of ROS (H_2_O_2_ and O_2_^•−^) as the key redox signaling agents, which are generated under the control of growth factors and cytokines, prominently including nicotinamide adenine dinucleotide phosphate (NADPH) oxidases on the plasma membrane, mitochondria, and the mitochondrial electron transport chain (ETC).^[^ ^60^ ^]^ Hydrogen peroxide (H_2_O_2_) is produced from O_2_ mainly through NADPH oxidases in conjunction with superoxide dismutases (SOD1‐3).^[^ ^61^ ^]^ Superoxide (O_2_^•−^) as a major source of H_2_O_2_, catalyzed by superoxide dismutases to H_2_O_2_ and O_2_. O_2_^•−^ is produced by specific enzymes, including ETC or membrane‐associated NOXs^[^ ^62^ ^]^ via single electron reduction of O_2_.^[^ ^63^ ^]^ Hydroxyl radical (•OH) is the most reactive and the most toxic ROS known.^[^ ^64^ ^]^ It is formed by the Fenton reaction between H_2_O_2_ and O_2_^•−^, driven by a Fenton reaction using iron (Fe^2+^, Fe^3+^) as the catalyst. The raised •OH leads to the increase of cytoplasmic viscosity and the accelerated formation of lipid peroxidation in ferroptosis.^[^ ^65^ ^]^

The amount of mitochondria‐targeting ROS generated is significant intermediates which derived from physiological processes, containing ETC and cell signaling. ROS surges disrupt the redox homeostasis, resulting in cell death via oxidative damage.^[^ ^66^ ^]^ Oxidative stress due to ROS surges is a key factor involved in DNA damage, cell signaling, cellular differentiation, and cancer proliferations and metastasis.^[^ ^67^ ^]^ Mitochondria sources ROS within the cell, which subsequently act on a plethora of cellular targets, thereby eliciting diverse biological activities. These activities are highly pleiotropic, including the regulation of cell death, stress sensor, inflammatory response, and metabolic adaptation as shown in Figure 3.^[^ ^68^ ^]^

Overloaded ROS level within mitochondria is a new paradigm for cancer treatment. Local overconcentration of ROS within mitochondria can release cytochrome c and other death‐related molecules into cytoplasm. This process will also cause serious damage to mitochondrial DNA (mtDNA) and reduce ATP and MMP synthesis, even inducing apoptosis.^[^ ^69^ ^]^ A significant number of novel nanomaterials have been developed to modulate mitochondrial ROS for cancer therapy. Therefore, we will give a detailed review in three parts (Table 1). (1) Redox homeostasis intervention strategy (RHIS)‐this part mainly focuses on modulating or intervening mitochondrial ROS level by nanotechnology; (2) Energy metabolism regulation strategy (EMRS), which takes advantage of the sensitivity of tumor cells to glucose level, and catalyzation of energy source glucose into toxic precursors H_2_O_2_; (3) Combination of these above related strategies in this section.

**TABLE 1:** Overview of some strategies with mitochondria‐targeting reactive oxygen species (ROS) modulation functions for cancer therapy

ROS modulation Strategy	Cancer therapy strategy	Mitochondria‐targeting unit	Advantage	Ref
Redox homeostasis intervention strategy (RHIS)	Glutathione (GSH) depletion and ROS generation	Triphenylphosphonium (TPP)	Mitochondrial oxidative stress amplifier	[59]
	Chemodynamic therapy (CDT), photodynamic therapy (PDT)	Ir (III) complexes	Magnetothermogenic nanozyme	[78]
	CDT, GSH depletion, and chemotherapy (CT)	TPP‐PEG_2K_‐LND	pH‐responsive nanoprodrug	[79]
Energy metabolism regulation strategy (EMRS)	Photothermal therapy (PTT), PDT, glucose oxidase (GOx)‐induced starvation	IR780 dye	Deep tumor penetration	[85]
	Sonodynamic therapy (SDT), GOx‐induced starvation	IR780 dye	Deep tumor penetration	[86]
Combination therapy	CT, PTT	IR780 dye	Mitochondria‐responsive drug release	[87]
	Radiation therapy (RT)	TPP	Nanoradiosensitizer to enhance RT effect	[88]
	RT, radiodynamic therapy (RDT)	Cationic Ru‐based photosensitizers (DBB‐Ru)	Cationic nMOFs for mitochondria targeting	[89]
	PTT, PDT	IR780	PFOB‐based nanoliposomes act as the “Nano‐RBCs”	[90]
	SDT, gas therapy	IR780	Reverse immunosuppression	[91]
	PTT, PDT	DPP2+	New strategy to design mitochondria‐targeting photosensitizers	[92 ]
	PDT	TPP	Near‐infrared (NIR) light‐activated PDT nanoplatform	[93]
	Photochemotherapy	Cationic Ru^2+^ complex	Near infrared‐assisted Fenton reaction	[94]
	PDT, GOx‐induced starvation	TPP	Self‐supply of O_2_ and H_2_O_2_ to enhance PDT effect	[95]

### 4.1 Redox homeostasis intervention

Cellular redox homeostasis is the balance between oxidizing species and reducing species.^[^ ^59^ ^]^ In normal cells, redox homeostasis is at physiologic levels, and plays vital role in many cellular processes. However, in cancer cells, overloaded metabolic demand results in an elevation of redox homeostasis, offering researchers a kind of strategy to induce cancer cell death. Recently, many researchers have utilized this redox homeostasis of mitochondria to accurately induce apoptotic cell death.^[^ ^70^ ^]^ Two major aspects, the ROS and antioxidants, divide this strategy into two ways: direct elevation of ROS levels and depletion of antioxidants (GSH).

NIR laser irradiation can directly trigger ROS in mitochondria. Wang et al. report a silica carbon hybrid nanoparticle coated by lipid membrane that targets mitochondria through pyruvate to produce ROS in mitochondria under NIR laser irradiation.^[^ ^71^ ^]^ Further, they identified the types of free radicals (such as O_2_^•−^; •OH), respectively with the electron paramagnetic resonance and fluorescence spectroscopy. Kim and co‐workers developed a mitochondria‐targeted NIR‐absorbing cryptocyanine probe (Mito‐Ccy), which could generate endogenous ROS with local heating.^[^ ^23^ ^]^ They point out that an activated electron transport chain is the requirement for photothermal therapy (PTT)‐mediated ROS generation in mitochondria. In addition, the photothermal effect of Mito‐Ccy results in its photoaction. Many other photochemical therapeutic agents that are involved in ROS generation could also originate in the mitochondrion.^[^ ^72^ ^]^ Recently, Wang and co‐workers developed a redox stimuli activatable metal‐free photosensitizer (aPS), which was connected to TPP to obtain ^mito^aPS and targeted mitochondria.^[^ ^73^ ^]^ The synthesized ^mito^aPS had dual responsiveness to both H_2_O_2_ and GSH. When attacked by exogenous H_2_O_2_, aPS reacted with GSH within mitochondria and was converted into a fluorescent molecule, but it itself would not be attacked by GSH. Upon two‐photon excitation, ^mito^aPS generated ^1^O_2_ and O_2_^•−^.

**FIGURE 4:**
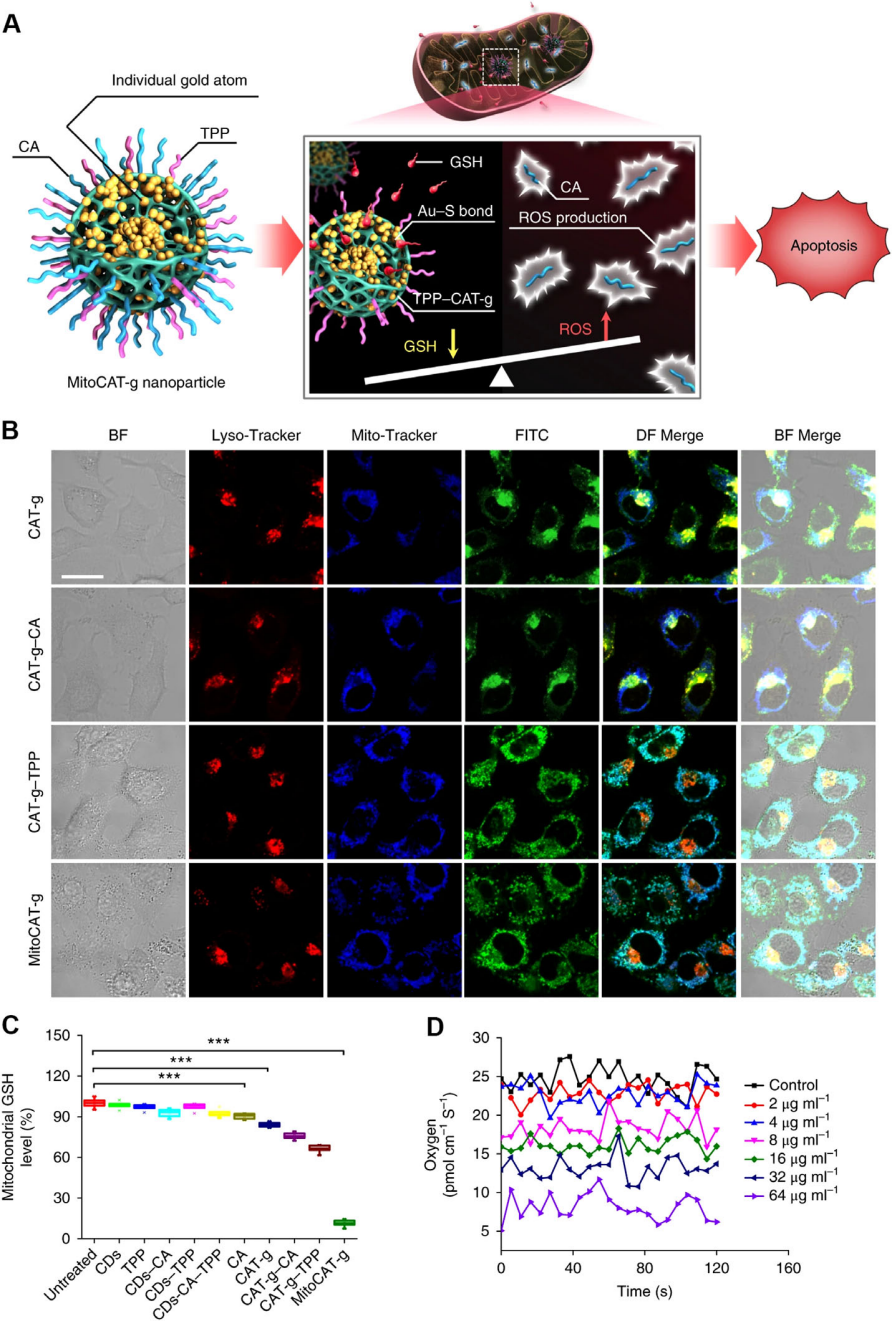
Nanotechnology strategies for mitochondria‐targeting reactive oxygen species (ROS) regulation. (A) The mechanism of MitoCAT‐g amplifies cellular oxidative stress within mitochondria. ROS generator (MitoCAT‐g) is developed from carbon‐dot‐based atomically gold (CAT‐g), and surface modifications of TPP and CA molecules are for mitochondrion‐targeting and ROS‐generating. (B) Confocal laser scanning microscopy (CLSM) results of HepG‐2 cells incubated with a different group for 2.5 h. Mitochondria and lysosomes are stained with blue (Mito‐Tracker) and red (Lyso‐Tracker), respectively. (C) Glutathione (GSH) levels within mitochondria after incubation with different nanoparticles. (D) Oxygen flux within single HepG‐2 cells after incubated with different concentrations of ROS generator (MitoCAT‐g). Reproduced with permission.^[^ ^59^ ^]^ Copyright 2019, Nature Publishing Group. BF, bright field; DF, dark field.

Overloading mitochondrial ROS level is the most effective way to influence the cellular redox homeostasis. However, it is difficult to generate toxic levels of ROS in mitochondria, as the elevated ROS can be quickly neutralized by the high levels of GSH in tumor cells. Thus, an ideal strategy for regulation of mitochondrial redox homeostasis is to simultaneously elevate ROS and deplete GSH.^[^ ^74^, ^75^, ^76^, ^77^ ^]^ Gong et al. developed a mitochondrial oxidative stress amplifier, MitoCAT‐g, which was constructed from carbon‐dot‐supported atomically dispersed gold for cancer treatment (Figure 4A).^[^ ^59^ ^]^ With the modification of TPP molecules on the surface, they target MitoCAT‐g to mitochondria with great specificity, and the data in Figure 4B by confocal laser scanning microscopy (CLSM) show that MitoCAT‐g group is ∼ 68% co‐localization with mitochondria. The atomically dispersed metal atoms provide the highest atom utilization and thus expose the most active sites for Au‐S chemical reaction, which enhances the GSH depletion property of MitoCAT‐g in cells. As shown in Figure 4C, MitoCAT‐g treatment causes significant reduction of the mitochondrial GSH to ∼ 12%, compared to the GSH content of 87% for CAT‐g group (carbon‐dot supported atomically dispersed gold) and 70% for CAT‐g‐CA (non‐specificity oxidative stress amplifier). One the other hand, the cellular ROS level is visualized by a fluorescent probe (DCFH‐DA). MitoCAT‐g group reveals a bright green signal, indicating that cinnamaldehyde molecules can effectively generate ROS within mitochondria. The JC‐1 dye and oxygen flux experiments demonstrate that MitoCAT‐g group greatly lowers the MMP and decreases the oxygen uptake, and lead to cell apoptosis (Figure 4D). Furthermore, MitoCAT‐g significantly inhibits tumor growth both in subcutaneous and orthotopic HCC‐PDX models in vivo, and demonstrates a negligible systemic toxicity for tissue and organ. It is therefore reasonable to conclude that the strategy of amplifying the oxidative stress in mitochondria by MitoCAT‐g could be a promising agent for anticancer applications.

Shen et al. developed a mitochondria‐targeting nanozyme (Ir@MnFe_2_O_4_) and achieved the depletion of GSH and generation of ROS, respectively.^[^ ^78^ ^]^ In their system, iridium complex plays a role as mitochondria targeting unit. Meanwhile, the intracellular GSH reduces Fe (III) to Fe (II), which can catalyze the H_2_O_2_ to high‐toxic •OH, as well as the reduction reaction of iron effective depletion of GSH. And the magnetic hyperthermia effects of inner MnFe_2_O_4_ nanoparticles disrupt the cellular redox homeostasis. Wang et al. constructed a novel nanoprodrug (BDTLAG) to enhance antitumor efficacy.^[^ ^79^ ^]^ At first, the BDTLAG nanoparticlesas are enriched in tumor region through passive targeting. When responsive molecule is released, the nanoparticles enter the MCF‐7 cells by active targeting. Meanwhile, as the pH and GSH‐responsive nanoprodrug release and GSH depletion, the endogenous ROS burst to break mitochondria redox homeostasis. However, the RHIS cannot completely eliminate cancer cells, due to the tolerance of high level of ROS. Thus, the “all in one” nanoprodrug can effectively enhance antitumor efficacy.

### 4.2 Energy metabolism regulation

ROS homeostasis is intrinsically linked to energy metabolism, such as pentose phosphate pathway for the NADPH supply.^[^ ^80^ ^]^ The physiologically relevant ROS (O_2_^•−^ and H_2_O_2_) in cells mainly derive from two pathways, NADPH oxidases or the mitochondrial electron transport chain.^[^ ^68^ ^]^ ROS, in particular H_2_O_2_, are able to reversibly oxidize critical, redox‐sensitive cysteine residues on target proteins. These oxidative post‐translational modifications regulate the biological activity of numerous enzymes and transcription factors.^[^ ^60^ ^]^

“Warburg effect” discloses that cancer cells undergo aerobic glycolysis, which refers to the fermentation of glucose to lactate in the presence of oxygen as opposed to the complete oxidation of glucose to fuel mitochondrial respiration.^[^ ^81^ ^]^ Compared to normal cells, glucose demand is high for cancer cells. GOx can efficiently consume glucose energy source and produces H_2_O_2_ within the same time. GOx has been strategically designed within various nanocatalytic systems to provide an alternative noninvasive method for cancer therapy.^[^ ^82^, ^83^, ^84^ ^]^ However, because of the continuous oxygen and glucose nutrient supply, it is hard to completely eliminate tumors with GOx alone. Generally, GOx mediated nanocatalyst requires to combine with extra therapeutic means to eliminate tumors.

**FIGURE 5:**
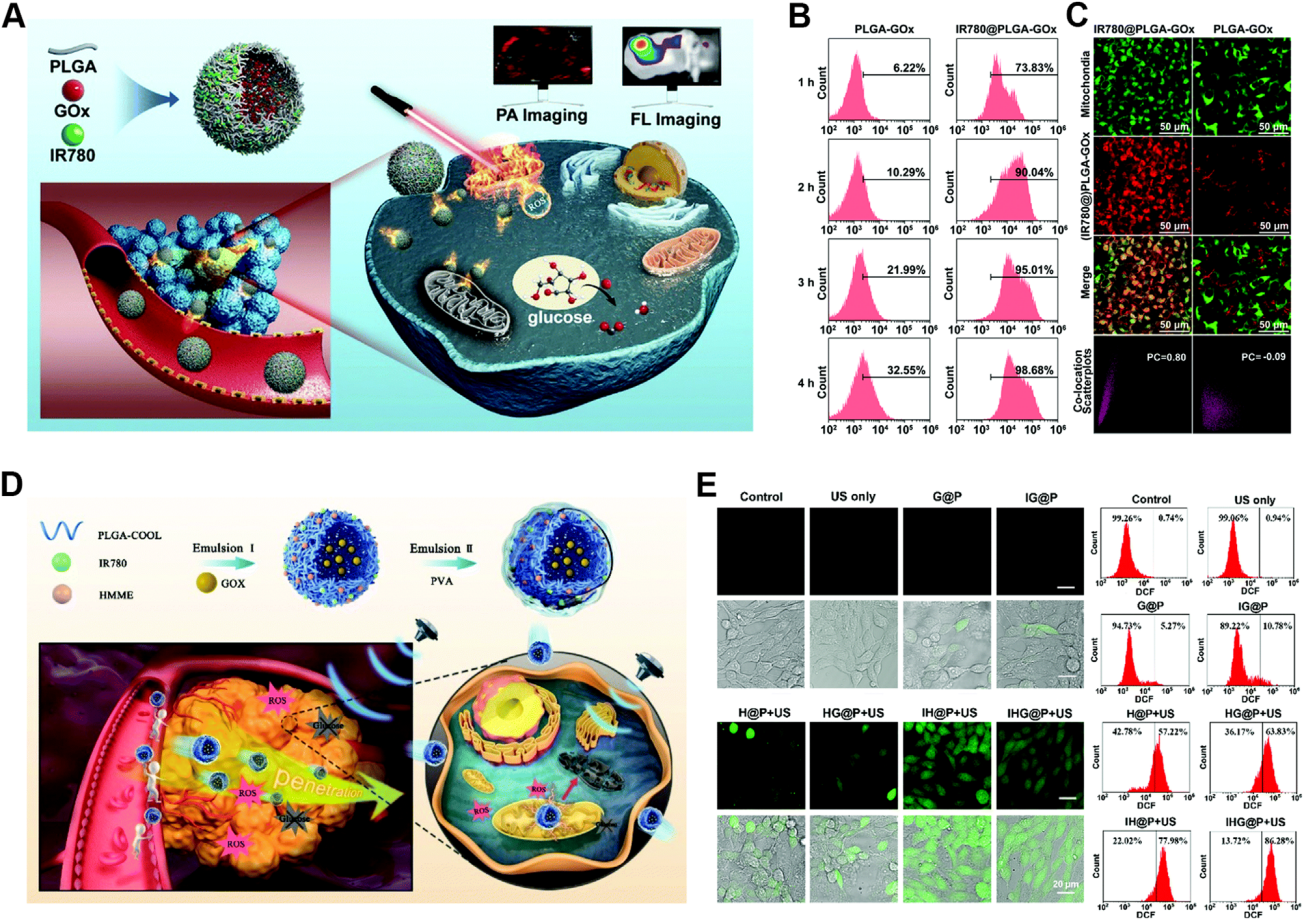
Examples of mitochondria‐targeting nanomaterials that manipulate and respond to glucose oxidase. (A) Scheme of the preparation of IR780@PLGA‐GOx nanoplatform for mitochondrial targeted therapy (phototherapy combined with enzyme (GOx)‐induced starvation therapy). (B,C) Intracellular uptake of different nanoparticles with various incubation times and the corresponding flow cytometry analysis results and confocal laser scanning microscopy (CLSM) results to verify the colocalization within mitochondria. Reproduced with permission.^[^ ^85^ ^]^ Copyright 2020, RSC Publishing Group. (D,E) The fabrication and mitochondrial‐targeted synergistic therapy (sonodynamic therapy (SDT)/starvation) of shell‐core nanostructured of IHG@P. Reproduced with permission.^[^ ^86^ ^]^ Copyright 2020, RSC Publishing Group.

IR780, a NIR imaging molecule, has exhibited notable characteristics as a photothermal conversion agent and photosensitizer, due to its high photostability and photothermal conversion efficacy. However, the phototherapy alone (PTT or PDT) is difficult to eliminate tumors and may result in recurrence. Wang et al. designed a strategy to combine the natural performances of IR780 (mitochondria‐targeting ability and good phototherapy effect) and GOx (energy metabolism interference by glucose consumption), achieving synergistic effect of phototherapy and GOx‐mediated energy metabolism interference.^[^ ^85^ ^]^ As shown in Figure 5A. Poly lactic‐*co*‐glycolic acid (PLGA) core/shell structured nanocarriers are used to load IR780 and GOx (the hydrophobic IR780 and hydrosoluble GOx locate in the shell and core, respectively). The hydrophobic groups of the *N*‐alkyl side chain of IR780 endow it with the characteristic of lipophilic cationic to achieve specific mitochondria accumulation ability (Figure 5B,C). Besides, the IR780@PLGA‐GOx nanospheres can penetrate the deep region of tumor assisted by the lipophilicity of IR780. However, the control group PLGA‐GOx just lights the marginal area of the tumor. In vivo experiments verify that with NIR laser irradiation, mitochondria‐targeting phototherapy and GOx‐mediated energy consumption have been triggered. On the other hand, their therapeutic process can be monitored by photoacoustic and fluorescence dual imaging.

Since NIR laser energy decays with penetration depth, traditional NIR laser‐triggered energy metabolism interference (EMI) strategy subjects to penetration depth, limiting their further applications. Zhang et al. developed a mitochondria‐targeted nanoplatform to achieve deep penetration and enhanced synergistic SDT with GOx‐mediated starvation therapy.^[^ ^86^ ^]^ With the aid of IR780, the nanoplatform IHG@P preferentially accumulate into the mitochondria of tumor sites (Figure 5D,E). Upon US irradiation, the synergistic therapy exhibits a 4.7‐fold lower tumor growth in volume than the control group (without IR780 for mitochondria‐targeting). Generally, their study combines the SDT and EMI methods for providing mitochondria‐targeting anticancer therapy as a promising strategy. Furthermore, this integration of EMI strategy and extra therapeutic methods can also be explored to X‐ray activated ROS generation strategy and radiodynamic therapy (RDT).

To sum up, enzyme‐mediated starvation has aroused interest in the field of cancer therapy, and various combination strategies have been developed, such as the paradigm of starvation therapy and the further utilization of H_2_O_2_ generated from glucose (the detail of GOx‐mediated starvation to produce H_2_O_2_ for ROS regulation is in Section 4.3). Besides, the glucose consumption will induce hypoxia and the acidity of the tumor microenvironment, which will promote the cooperation of EMRS with hypoxia‐activated therapy and pH‐responsive drug release. However, there remain many challenges, such as the accuracy of delivery to cancer cells, and the biotoxicity to the normal tissues and cells.

### 4.3 Combination therapy

Due to the higher susceptibility of mitochondria than other subcellular organelle, a given amount drug and ROS enriched within mitochondria will cause much higher cytotoxicity, let alone that monotherapy strategy may lead to the emergence of drug resistance or high‐dose administration. Thus, plenty of combination strategies targeting mitochondria have been developed to improve therapy efficiency. For example, mitochondria‐targeting PTT is always closely followed with chemotherapy (CT) or PDT combination, especially for the organic molecules based photothermal conversion agents. The combination of CDT with mitochondria‐targeting ROS regulation also seems to be a perfect match, which takes advantage of high H_2_O_2_ concentration within mitochondria and translates the H_2_O_2_ to high toxicity •OH. Moreover, GOx‐mediated starvation is applied to the combination therapy to produce H_2_O_2_. Hence, we will review the recent literatures on mitochondrial‐targeted combination therapy.

**FIGURE 6:**
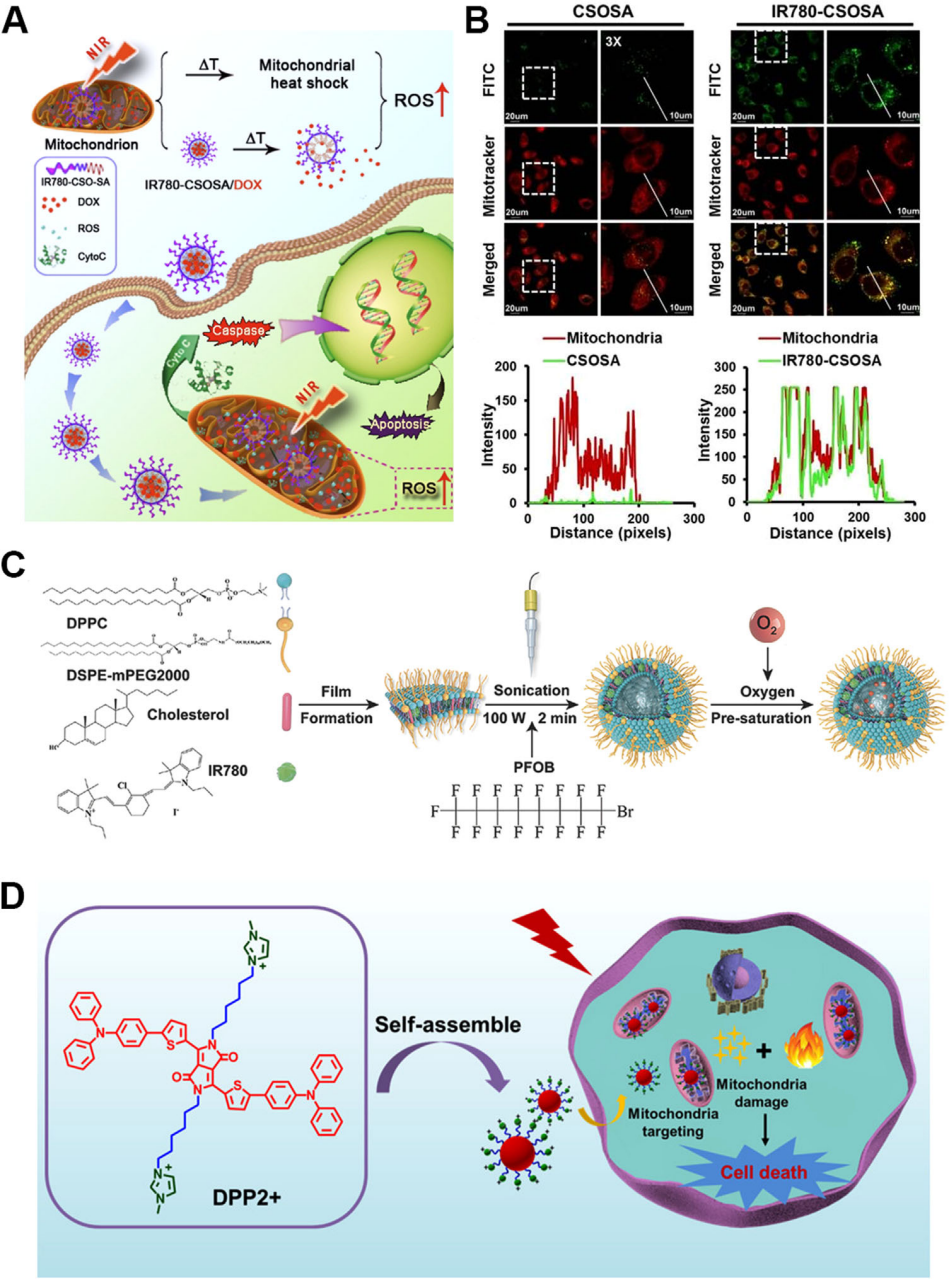
Combination therapy based on Mitochondria‐targeting nanomaterials in cancer. (A) The near‐infrared (NIR) triggered drug delivery system with elevated mitochondrial reactive oxygen species (ROS) level for cancer therapy. Reproduced with permission.^[^ ^87^ ^]^ Copyright 2019, Ivyspring International Publisher. (B) Cellular uptake and mitochondrial co‐localization of IR780‐CSOSA micelles in MCF‐7 cells, and the line scanning profiles of nanomaterials in the corresponding confocal laser scanning microscopy (CLSM) images. (C) Synthetic pathway of O_2_ self‐supply nanoplatform (PFOB@LIP‐IR780). Reproduced with permission.^[^ ^90^ ^]^ Copyright 2018, John Wiley & Sons Publishing Group. (D) Mechanism of mitochondria‐targeting DPP2+ nanoparticles for phototherapy (photodynamic therapy (PDT) and photothermal therapy (PTT)). Reproduced with permission.^[^ ^92^ ^]^ Copyright 2020, ACS Publishing Group.

CT is still the mainstream method to fight against cancer, especially that some drugs work on specific intracellular sites. Mitochondria are regarded as the major target. Efficient targeting and stimuli‐responsive drug release within mitochondria is a pre‐requisite for mitochondria‐targeted drug delivery. Tan et al. developed a mitochondria‐targeting glycolipid conjugates (CSOSA) nanoplatform (Figure 6A), which includes chemotherapeutic drug doxorubicin (DOX), photothermal and lipophilic agent IR‐780.^[^ ^87^ ^]^ In vitro CLSM co‐localization results demonstrate that IR780‐CSOSA micelles have a bright yellow fluorescence (merged by red and green fluorescence) after being incubated with MCF‐7 and HepG2 cells for 4 h (Figure 6B). Under NIR‐laser irradiation, DOX is released from IR780‐CSOSA micelles into mitochondria, which is verified by noticeably red DOX fluorescence. Besides, ROS levels of laser‐stimulated IR780‐CSOSA/DOX group are the highest, which is 2.85 folds of DOX, indicating a synergistic ROS evolution and elevation, and high levels of ROS promote the translocation of cytochrome c to cytosol, a significant apoptosis indicator. In vivo results demonstrate that the IR780‐CSOSA/DOX‐NIR is the most effective group for inhibiting tumor growth, 85.3% compared to 55.0% of DOX group. In addition, the mitochondria‐targeting chemo‐phototherapy combination group shows the longest median survival time with 100% survival rate and without tumor recurrence within 40 days.

Radiation therapy (RT) is widely used for clinical antitumor applications. However, excessive dosage of ionizing radiation causes severe damage to normal tissues. Consequently, subcellular organelle targeting strategy may be an ideal choice with higher selectivity and efficiency. Li et al. reported a mitochondria‐targeting TiO_2_‐Au‐TPP nanoradiosensitizer to enhance their RT effect.^[^ ^88^ ^]^ Upon X‐ray radiation, the nanoparticles could produce ROS within mitochondria, which further induces a domino effect on ROS burst and leads to cell apoptosis. In vivo data demonstrates that the nanoradiosensitizer can prolong survival rate to 50 days. Besides, Ni et al. developed a Hf‐DBB‐Ru nanoparticels for mitochondria‐targeting RT and RDT.^[^ ^89^ ^]^ Under X‐ray irradiation, nMOFs efficiently produce •OH from metal‐nodes (Hf_6_ SBUs) and ^1^O_2_ from the DBB‐Ru organic linkers to result in RT‐RDT effects. Furthermore, RT‐RDT effectively depolarizes the mitochondrial membrane, inducing cell apoptosis. In vivo results also show that the average tumor volume of RT‐RDT group is only 3.0% of that of the PBS control group.

More impressively, mitochondria‐targeting PDT and PTT synergistic strategy will produce ROS and hyperthermia within mitochondria, amplifying the therapeutic effects. A mitochondria‐targeted nanoplatform (PFOB@LIP‐IR780 NPs) is developed for synergistic cancer phototherapy,^[^ ^90^ ^]^ which provides a novel strategy to achieve high therapeutic efficacy (Figure 6C,D). Compared with ICG molecules for phototherapy, IR 780 molecules are selected because of its easy encapsulation into the nanoliposomes, high ^1^O_2_ quantum yield (the value is 0.127), and intracellular mitochondria‐targeting ability. Cellular localization results show that the corresponding Pearson Correlation coefficient is 0.77 for mitochondria, which demonstrates that the PFOB@LIP‐IR780 selectively locate into mitochondria, and the relative cell viability in vitro verifies that PFOB@LIP‐780 holds the highest therapeutic efficacy. Besides, due to the PFOB, a component of oxygen carrier, PFOB@LIP‐780 shows a mitochondria‐targeting ability and O_2_ amplified PDT. Except regarding as a classical photosensitizer, IR780 is also a sonosensitizer for SDT. Ultrasound (US)‐irradiated nanosystem (PIH‐NO) is designed to reverse immunosuppression and enhance SDT effect.^[^ ^91^ ^]^ Due to mitochondria‐targeting ability of IR‐780, this nanosystem can effectively accumulate within mitochondria. Under the irradiation of US, PIH‐NO shows a burst O_2_ release, which increases the SDT effect and alleviates hypoxic TME. In addition, NO released from PIH‐NO reeducates M2 macrophages to M1 phenotype and remodels the immune microenvironment. In vivo results show that the DCs maturation is 36.5% of PIH‐NO+US group, which is 3.6‐fold of IH+US group, indicating a provoked immune response of PIH‐NO in vivo.

Xie group synthesized a mitochondria‐targeting photosensitizer diketopyrrolopyrrole (DPP2+) based nanoparticles,^[^ ^92^ ^]^ which can be used for photothermal enhanced photodynamic therapy (Figure 6D). The surface zeta potential of DPP2+ nanoparticles increases from −11.5 to 16.45 mV, which endows the DPP2+ nanoparticles the power to target mitochondria, and the colocalization coefficient of mitochondria is 0.73 for DPP2+ nanoparticles group. Under the 635 nm laser irradiation, the cytotoxicity in Hela cells is 90% with 5 μM DPP2+ incubation. Meanwhile, in vivo results show that the DPP2+@F127 group exhibited a significant inhibitory both intravenous and intratumor injection. Liu et al. reported a mitochondria‐specific NIR‐activated PDT nanosystem (Nd^3+^‐sensitized upconversion MOFs, named as UCMTs),^[^ ^93^ ^]^ and the Janus structures endow further asymmetric modification of TPP molecules. Then, with 808 nm NIR irradiation, an efficient resonance energy transfer from upconversion nanoparticle to porphyrinic‐based nMOFs domain, producing ROS within mitochondria.

**FIGURE 7:**
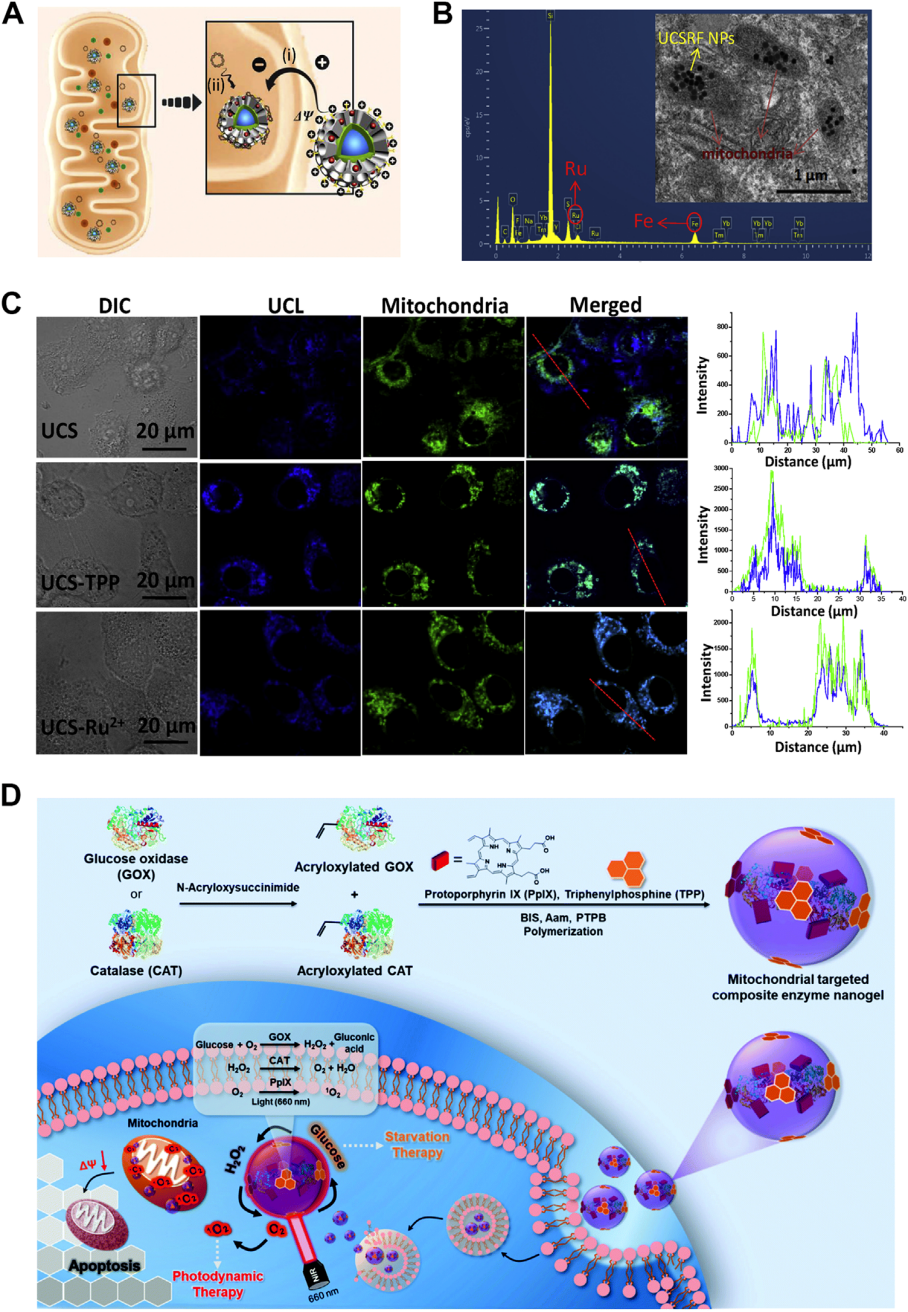
Mitochondria‐targeting nanomaterials for combination therapy. (A) Scheme of the Ru^2+^ complex‐mediated mitochondria targeting process. (B) Biological‐transmission electron microscopy (Bio‐TEM) to reveal the cellular mitochondria targeting effect of the Ru^2+^ complexes. (C) Confocal laser scanning microscopy (CLSM) results of HepG2 cells treated with different formulations to colocalize with mitochondria, and the blue signal represents the UCS, and the green signal is from Mito Tracker Green. Reproduced with permission.^[^ ^94^ ^]^ Copyright 2017, Elsevier Publishing Group. (D) The design of composite GOX‐CAT‐PpIX enzyme nanogels for synergistic starvation and photodynamic therapy. Reproduced with permission.^[^ ^95^ ^]^ Copyright 2021, John Wiley & Sons Publishing Group.

Chemodynamic therapy is a new strategy inspired by fenton reaction for environmental application. The essential parts of CDT for bio‐applications are endogenous H_2_O_2_ and Fenton agents (Fe, Cu, Mn). Noteworthy that the over expressed H_2_O_2_ around mitochondria is conducive to fenton reaction, however, the pH environment (∼ 8.0) of mitochondria is not suitable for Fenton catalytic reaction. Thus mitochondria‐targeted related literatures are few reported. However, combination CDT with other mitochondria‐targeting ROS regulation seems to be a gorgeous combination, which takes advantage of high H_2_O_2_ concentration within mitochondria and transfers the H_2_O_2_ to high toxicity •OH. Photo‐chemodynamic combination nanoplatform based on NaYF_4_: Yb^3+^, Tm^3+^@NaYF_4_@ dSiO_2_@mSiO_2_‐Ru^2+^/Fe^2+^ core/shell UCSRF nanoparticles is shown in Figure 7A.^[^ ^94^ ^]^ In their work, Ru^2+^ complexes on UCSRF nanoparticles are a kind of lipophilic cations, which can target the inner membrane of mitochondrial (Figure 7B,C). Different from the traditional Fenton reaction, these photo‐chemodynamic strategy utilizes UV light, which comes from the NIR light‐upconverted to sustain the Fenton reaction (H_2_O_2_+*hv*→2(•OH)). In vivo experiments verify substantial tumor regression in UCSRF+NIR laser group, meanwhile, neither any abnormal behavior nor significant weight loss.

Although chemodynamic therapy has achieved great triumphs for cancer therapy, low concentration of endogenous H_2_O_2_ is still a huge challenge for their biomedicine. Mitochondrial of cancer cells have an altered redox homeostasis, such as high levels of H_2_O_2_. Luo et al. designs a mitochondrial targeting nanogel bioreactor, which is composed of GOx, catalase (CAT), photosensitizer (protoporphyrin IX, PpIX), and TPP molecules (Figure 7D).^[96]^ Due to the mitochondria‐targeting TPP molecule, the nanogel can efficiently target the mitochondria of 4T1 cancer cells, transforming intracellular glucose to generate H_2_O_2_ by GOx, and H_2_O_2_ is further degraded to generate O_2_ by CAT. Moreover, the generated O_2_ serves as an enhancer of GOx catalysis efficiency as well as the source for PDT. The in vitro results show that combination of starvation and photodynamic therapy has the best anticancer effect.

In conclusion, combination strategy for mitochondria‐targeting has been applied to many monotherapies, such as combination of PDT and PTT, CDT and PDT, RT, and RDT. The major mechanism originated from the enrichment property of energy and ROS. However, the existing combination strategies also face the challenge that modulation the suitable environment for each monotherapy of the combination strategies.

## 5 CONCLUSION AND PROSPECT

As a metabolism subcellular organelle, mitochondria play an important function in cellular signaling pathways, proliferation, and apoptotic cell death, which makes it to become an important target. In this review, we summarize the structure, functions of mitochondria, and mitochondria‐targeting strategies. As such, we argue that the TPP (positive charge)‐related modifications are major ways in these fields. It remains difficult to effectively accumulate ROS within mitochondria, and the development of higher ROS concentrations within mitochondria through nanomaterials is ongoing. Thus, nanomaterials for mitochondria‐targeting ROS modulation have been summarized, such as CT, PTT, PDT, CDT, RT, and combination therapy. Based on the recent research, we assert that the main direction of mitochondria‐targeted nanomaterials with ROS generation property is rooted in affecting energy metabolism, and disturbing redox homeostasis, yet there still remains a great demand for additional forward progress in this important area.

In the future, many challenges are left for further application of mitochondria‐targeting strategy. First, mitochondria‐targeting CT is proven to be the most effective strategy, which kills cancer cells through damaging mtDNA and inducing apoptosis. However, the biocompatibility of chemotherapeutic drugs shall be considered. By contrast, ROS generated strategies for mitochondria‐targeting hold a good potential to reduce toxicity. Second, the specific mitochondria‐targeting units are also needed for better therapeutic effect on one hand, the suitable CDT for mitochondria environment can be designed on the other hand, such as Mn‐based Fenton agents. Third, the preparation of nanomaterials must be extremely smart with tunable mitochondria‐targeting activity for precision therapy. Fourthly, comprehensive evaluation and long‐term monitoring of the realistic effects on tumor are needed, especially for further analysis of the metabolic mechanism and metabolic pathway in vivo to promote their potential clinical transitions. Fifthly, the applications of mitochondria‐targeting strategy shall be expanded to mitochondria‐related diseases, such as cardiovascular diseases and neurological diseases. Overall, although the problems in developing mitochondria‐targeting nanomedicine with ROS generated properties, researchers from various fields should collaborate more frequently to conduct in‐depth research and drive further development and even clinical translation.

## CONFLICT OF INTEREST STATEMENT

The authors declare no conflicts of interest.
